# Evaluation method of ore grade estimation effectiveness

**DOI:** 10.1371/journal.pone.0309696

**Published:** 2024-09-12

**Authors:** Zhan-Ning Liu, Chuan-Lei Lu, Rui Tian, Yang-Yang Deng, Zhan-Hui Liu, Peng-Wei Zhang

**Affiliations:** 1 Anyang Institute of Technology, Anyang, Henan Provence, The People’s Republic of China; 2 Harbin Natural Resources Comprehensive Investigation Center of China Geological Survey, Harbin, Heilongjiang Provence, The People’s Republic of China; Atlantic Technological University, IRELAND

## Abstract

This study proposes a new method to evaluate the effectiveness of orebody grade estimations, drawing upon the analysis of existing evaluation methods for grade estimation. This new approach addresses factors such as uneven sampling and asymmetric estimation range, which are challenging to overcome with existing evaluation techniques. The core principle of this method involves documenting how frequently individual samples are used during grade estimation and calculating the total distance weights for each sample. Subsequently, the usage frequency and total weight of the samples are standardized, and these standardized values are weighted based on the sample grades. A comparison is made between the weighted sample grades and the estimated grades, with the closeness between the two serving as a metric for assessing the effectiveness of the estimation. This study compares the new evaluation method to the direct comparison and cross-validation methods, examining the effectiveness of grade estimation using the inverse distance weighting (IDW) method. The findings revealed that: (1) The new evaluation method theoretically accounts for the systematic deviation between the statistical measures of estimated and sample grades resulting from uneven sample distribution, offering a fresh approach for enhancing the effectiveness of orebody grade estimation. (2) In the grade estimation of experimental Fe samples, the frequency of usage and the sum of distance weights were unequal. This inequality significantly contributes to the systematic deviation between the estimated and sample grades. (3) Comparing the new evaluation method to others confirms the stability and reliability of the new approach for evaluating the effectiveness of orebody grade estimation. This novel method demonstrates theoretical advantages and practical utility. (4) The deviation between the estimated grades and the statistical results of sample grades is influenced by the distribution pattern of sample grades, the spatial relationship between samples and estimation blocks, and the inherent systematic error associated with the IDW method. This systematic error cannot be overlooked.

## Introduction

Estimating ore grade is pivotal in mining exploration and development, as it directly impacts the economic value of mineral resources and the scientific rigor of mining decisions. With the depletion of mineral resources and the increasing challenges in exploration, efficiently and accurately estimating ore grade has emerged as a critical issue requiring urgent resolution within the mining industry. The evaluation methodologies for ore grade estimation effectiveness are integral to the precision of orebody modeling and significantly influence the subsequent efficiency and economic returns of mineral resource development and utilization. Hence, exploring scientific and rational evaluation methods for ore grade estimation effectiveness is paramount for enhancing mineral resource exploration’s accuracy and economic feasibility.

In research on the evaluation of ore grade estimation effectiveness, scholars have conducted extensive studies and proposed many evaluation methodologies. These methods can be divided into five groups: First, comparing the statistical characteristics of estimated grades to sample grades to assess estimation effectiveness [1,2]. However, this approach is constrained by issues such as uneven sample distribution and irregular orebody morphology in exploration projects, making it challenging to obtain optimal estimated grades [3–5]. Second, utilizing multiple estimation methods to cross-validate each other to determine the accuracy of estimated grades [6,7]. Although this method can verify the reliability of new ones to some extent, the optimal estimated grades identified are often approximations [8–10]. Third, directly comparing estimated grades to true grades offers the best evaluation effect but is limited in application due to the difficulty in obtaining true grade data [11–13]. Fourth, assessing estimation effectiveness through variogram analysis [14] emphasizes the spatial structural consistency between estimated and sample grades, albeit with slightly inferior accuracy [15–17]. Lastly, cross-validation methods have emerged as the mainstream approach for verifying grade estimation effectiveness by comparing estimated grades at sample points with actual sample grades [18,19].

Since these methodologies have played a significant role in evaluating the effectiveness of ore grade estimation, they still exhibit shortcomings and limitations. For instance, the direct comparison of estimated grades to sample grades struggles to overcome systematic biases inherent in exploration projects. Although cross-validation among multiple methods can enhance estimation reliability, determining optimal estimated grades remains an approximation. The difficulty in acquiring true-grade data constrains the application of direct comparison methods. Despite the variogram approach emphasizing spatial structural consistency, its accuracy still leaves room for improvement. To some extent, cross-validation methods address the impact of sample distribution and orebody morphology on estimation results, but the optimal estimation parameters identified cannot fully equate to the globally optimal results for grade estimation.

Accordingly, the research explores novel evaluation methods for ore grade estimation effectiveness to enhance the accuracy of estimated grades. Specifically, it integrates the strengths of existing methodologies while attempting to introduce new evaluation indicators and algorithms to develop a more scientific and rational approach for assessing the effectiveness of ore grade estimation. This methodology will enable a more precise evaluation of the advantages and disadvantages of different estimation methods and provide a more reliable grade distribution model for exploring and developing mineral resources.

This research addresses the inaccuracies inherent in current evaluation methods for ore grade estimation effectiveness and proposes a more efficacious approach. In addition, it validates the feasibility and effectiveness of the proposed method through empirical analysis, offering novel insights and technical support for grade estimation efforts within the mining industry.

This research holds significant theoretical implications and vast potential for practical applications. By exploring novel evaluation methods for ore grade estimation effectiveness, it not only enhances the accuracy and economic efficiency of mineral resource exploration but also contributes to the sustainable development of the mining sector with renewed momentum.

This paper is structured to address the effectiveness of orebody grade estimation comprehensively. It begins with an Introduction outlining the need for such evaluation. The Methods section explores established and novel approaches focusing on sample usage and distance weights. The Orebody Models and Data Preprocessing section lays the geological foundation and data preparation. Experimental Methods and Results illustrate the practical application and analysis. The Analysis of Sample Usage introduces the weight standardization method. The Evaluation section discusses the implications of the new method, culminating in a Discussion, Conclusion, and Acknowledgments.

## Methods for grade estimation and evaluation

### Grade estimation method

Inverse Distance Weighting (IDW) is a common spatial interpolation method. The main steps of IDW are as follows:

(1) Determine the spatial positions of the estimated block and known samples

The first step is data preparation. The content includes the spatial coordinates and range of the block to be estimated, as well as the coordinates and grade of the known samples. This study established a grade database and a 3D model of the orebody using 3D mining software. The sample grade database contains spatial and grade information on the sampling locations. The three-dimensional model of the orebody was divided into blocks, and the spatial position of the center point of each block was used as the estimated position for the grade.

(2) Weight calculation

The smaller the distance between the sample and the position of the block to be estimated, the greater the impact of the sample grade on the estimated grade. Therefore, the weight should be inversely proportional to the distance. The following formula can be employed to calculate the inverse distance weight:

$$w_{ij}=\frac{\frac{1}{{\left(d_{ij}\right)}^{p}}}{\sum_{i=1}^{N}\frac{1}{{\left(d_{ij}\right)}^{p}}}$$ (1)

where *w*_*ij*_ is the weight of the i-th sample point that participated in the estimation when valuing the j-th target point; *d*_*ij*_ is the distance between the i-th sample and the estimated j-th position; *P* is an adjustable parameter, and in this study, 2 was used.

Weighted Solution Calculation

The weight of each sample participating in the estimation of *P*_*j*_ for the *j*-th block was calculated. The distance weight was used for sample grade weighting, and the grade of the block was estimated. Among them, *N* is the number of samples involved in estimating the *j*-th block. The grade of the j-th sample participating in the estimation is *z*_*i*_, with a weight of *w*_*ij*_. Formula (2) can be employed to calculate ore grade [20,21]:

$$P_{j}=\frac{\sum_{i=1}^{N}w_{ij}\times z_{i}}{\sum_{i=1}^{N}w_{ij}}$$ (2)

### General methods for estimating the effectiveness of the evaluation

(1) Direct comparison method

The estimated block grade is directly compared to the combined sample grade. The estimation parameters are deemed optimal when they closely match the grade of the combined sample. Typically, the primary statistical results of the block estimated grade and combined sample grade serve as the parameters for comparison.

(2) Cross-validation method

First, the grade of the block closest to the sample is estimated, and then the estimated grade is compared with the combined sample grade. The estimated parameter closest to the sample grade is considered the optimal estimation parameter. Similarly, the statistical results of the estimated grade are compared to those of the combined sample grade.

### New methods for estimating the effectiveness of the evaluation

(1) Sample usage frequency for weighting

The number of times a sample is used during the estimation process is counted. The number of samples is utilized to weigh the sample grade. The weighted sample grade is used for grade estimation comparison. The weighted grade is compared to the estimated grade results. In addition, the effectiveness of the orebody grade estimation is evaluated. Formula (3) is applied to calculate the number of times the sample.

$$\lambda_{\text{N}i}=\sum_{j=1}^{M}N_{ij}$$ (3)

where λ_*Ni*_ is the number of times the i-th sample is used; *N*_*ij*_ is the number of times the i-th sample is used for estimating the j-th block; M is the total number of blocks to be estimated; N is the total number of samples.

(2) Weighting of Sample Distance Weights

When IDW is used for block grade estimation, the distance weight between the sample and the block to be estimated is calculated. The weights of the samples involved in the calculation are recorded, and the total weight of a single sample in the grade estimation is determined, as shown in formula (4). The weight of the sample is standardized to obtain the distance weight of the sample, which is still called the distance weight. The sample distance weight is employed to weigh the sample grade. The weighted grade is compared to the estimated grade and used to analyze the effectiveness of the grade estimation.

$$\lambda_{\text{W}i}=\frac{\sum_{j=1}^{M}w_{ij}}{\sum_{j=1}^{M}\left(w_{1j}+w_{2j}+\ldots +w_{\text{N}j}\right)}$$ (4)

where λ_*Wi*_ is the weight of the distance weight of the i-th sample; *w*_*ij*_ is the distance weight of the i-th sample when estimating the j-th block.

## Orebody models and grade data preprocessing

This study used an orebody as a case for grade estimation. Based on existing geological data, a three-dimensional model of the orebody described below was created by analyzing geological conditions and the orebody’s occurrence characteristics. Grade statistics were executed, and sample grades were combined. These composite samples served as the foundational data for the subsequent grade estimation and sample grade comparison.

### Geology of the research area

Magmatic rocks are distributed within the mining area investigated in this study, with Quaternary (Q) loose layers scattered across the region. These layers are primarily concentrated in lower-lying areas and valleys, comprising residual slope deposits and alluvial-proluvial layers. The primary lithology comprises brownish-yellow, yellow, maroon, and gray-black humus soil, sub-sandy clay, sub-sandy soil, and gravelly sandy soil.

A compresso-torsional fault (Fault F1) trending northeast is exposed in the mining area towards the northeast of Dagang Forest Farm. This fault cuts through the mining area at the center, with a strike trending northeast, a dip direction of 130° to 140°, and a dip angle of 70° to 80°. It extends approximately 10 kilometers within the survey area beyond the map at both ends. Geomorphologically, it forms a linear intermountain valley. A small compresso-torsional fault (Fault F3) trending north-north-east is exposed in lines 16–12 of the region, dipping towards the west with an approximate dip angle of 75°, interrupting the Li-1 orebody, causing it to move 170 meters north-south and 80 meters vertically.

### Three-dimensional model of the orebody

**Fig 1:**
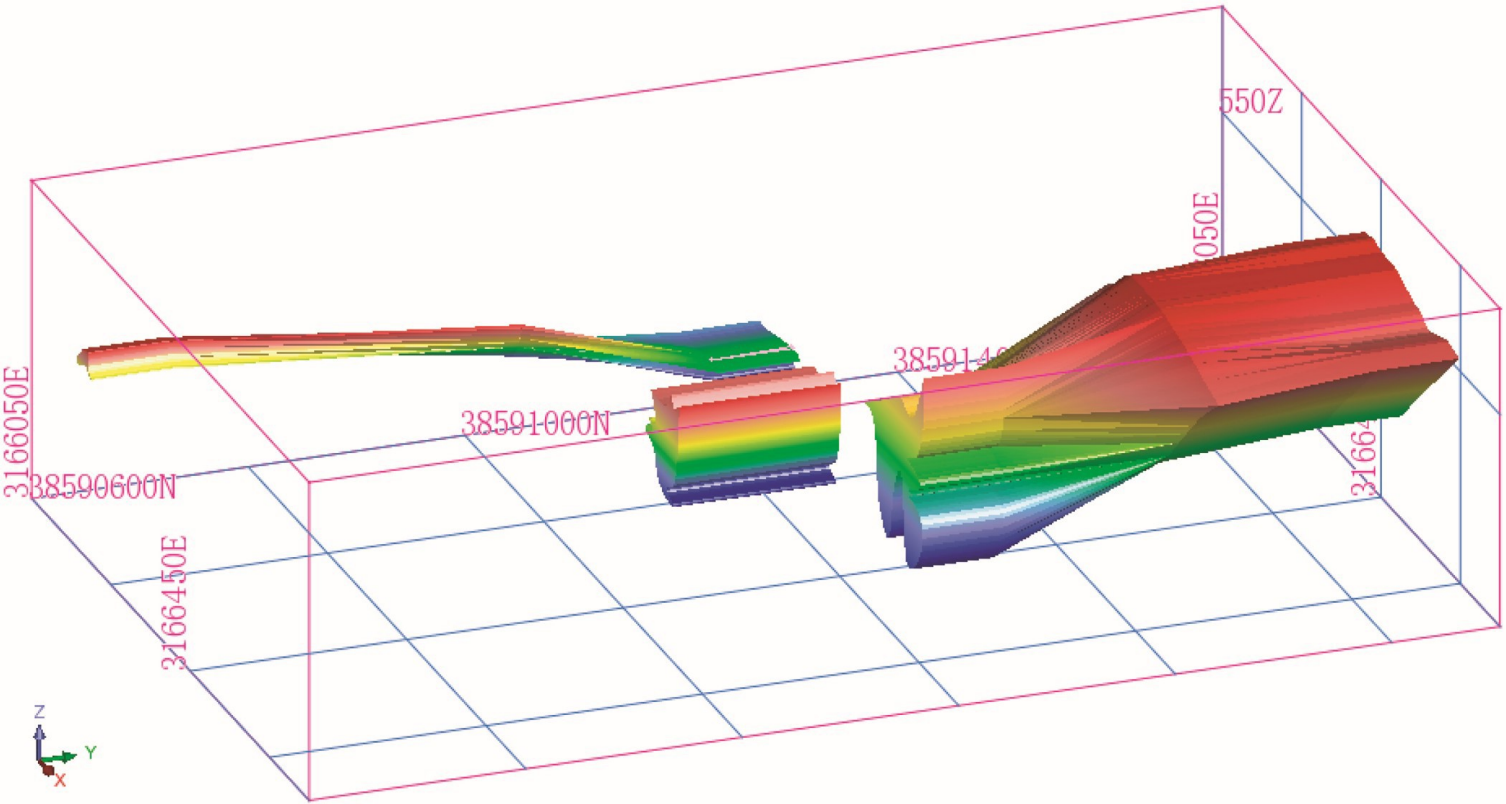
Three-dimensional model of the orebody.

**Fig 2:**
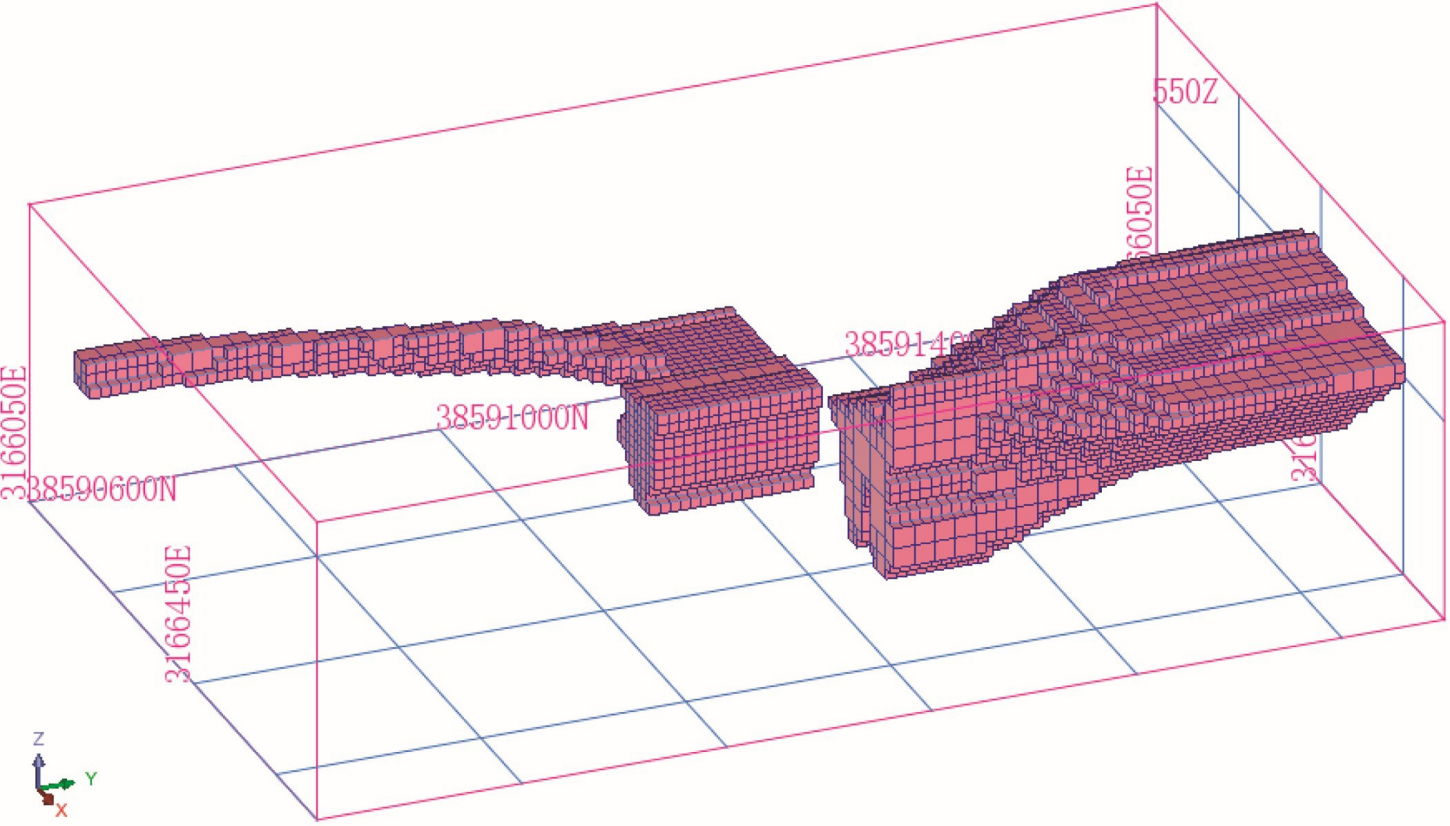
Block model.

A three-dimensional orebody model of the mine was constructed using the existing 2D profile of the orebody and orebody data, as shown in Fig 1. The interior of the orebody is divided into blocks to estimate the orebody grade. The block shape is rectangular, as shown in Fig 2.

### Sample grade data preprocessing

**Fig 3:**
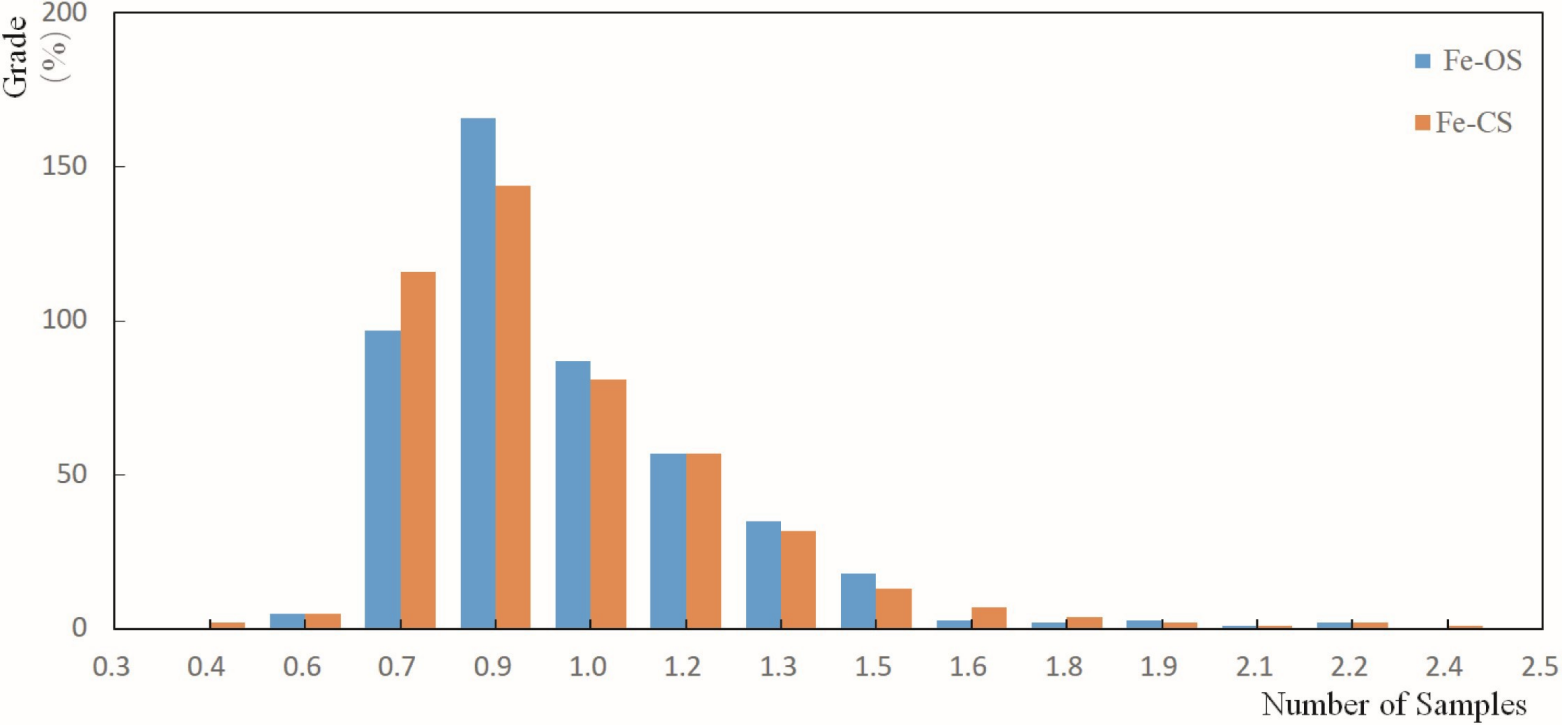
Distribution of the original and combined samples.

In order to make the spatial distribution of the original Fe sample more uniform, grade combinations were performed on the original sample based on the sample’s sampling length to obtain a composite sample. The distribution pattern of the grade before and after the sample combination is shown in Fig 3. The sample grades before and after the combination were statistically analyzed, and the statistical results are listed in Table 1. The average grade of the combined samples is slightly reduced compared to the original sample grade. The sample combination resulted in an average grade. Therefore, the standard deviation of the combined sample grade decreased.

**Table 1:** Statistics of the original and combined samples.

Sample type	Minimum	Maximum	Mean	Standard Deviation	Median	Kurtosis	Skewness
Original	0.25	2.3	0.899	0.2647	0.85	1.6017	4.2676
Combined	0.25	2.1693	0.8903	0.2481	0.8311	1.449	3.7526

## Experimental methods and estimation results

The results of the direct comparison and cross-validation methods were compared to those of the new method to validate the new grade estimation evaluation method. The new grade estimation evaluation method is based on the IDW method to demonstrate its effectiveness.

### Experimental design and steps

**Fig 4:**
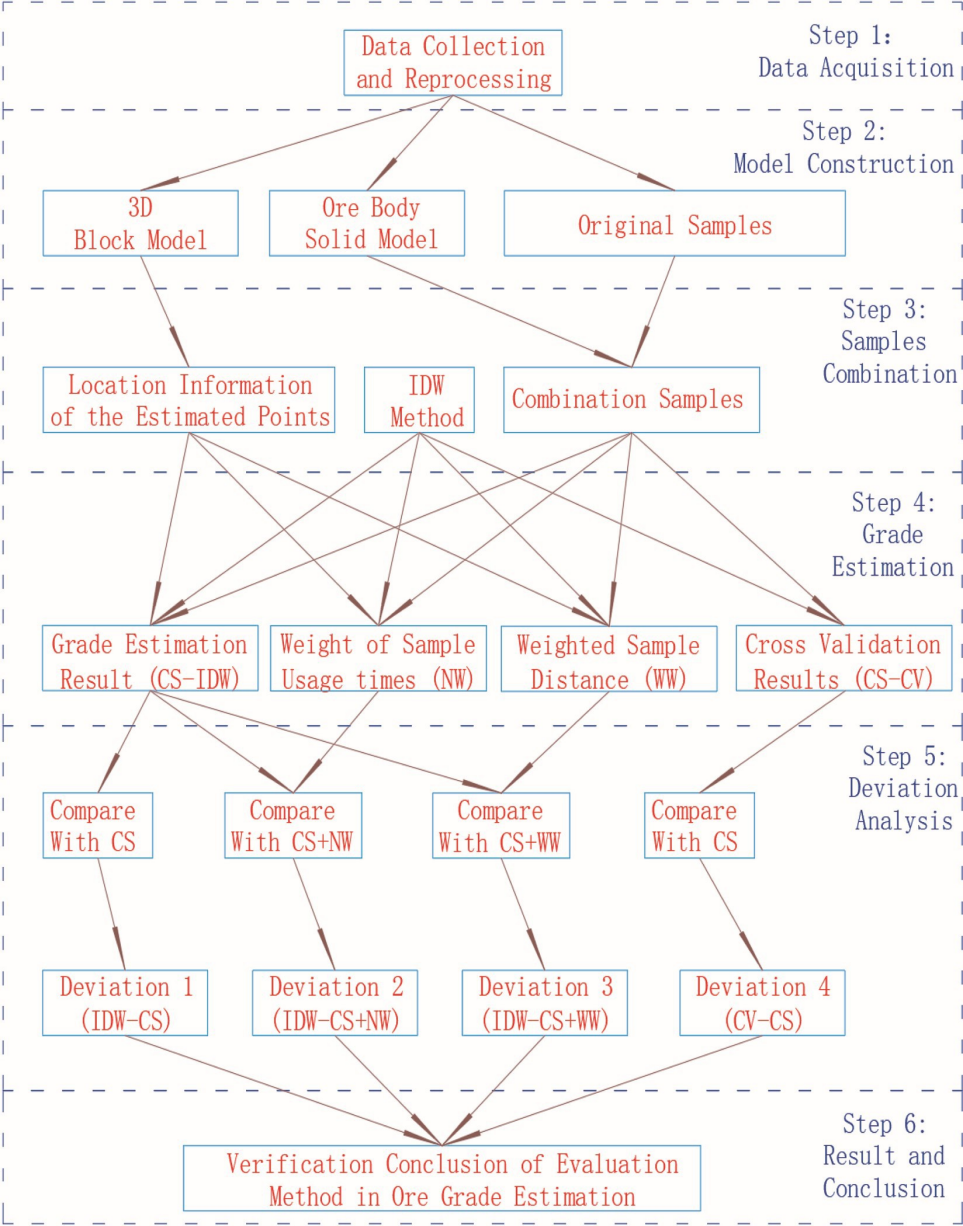
Experimental flowchart.

This study mainly focused on six aspects: data preparation, configuration construction, sample combination, grade estimation, estimation effect analysis, research results, and conclusions. The research steps and work content are detailed in Fig 4. The main steps and critical experimental parameter settings are briefly described as follows:

(1) Data preparation

A three-dimensional model of the orebody was constructed, and the interior of the three-dimensional orebody was divided into blocks. Each block formed an estimated grade carrier. The block spatial coordinates were output in the text format for MATLAB calculations.

The geological database was constructed based on drilling hole data, laboratory data, lithology data, inclinometer data, and other information obtained from exploration engineering. The original sample grade underwent statistical analysis, and the results are detailed in Table 1 and Fig 3. The combination of sample grades considered the original sample grade and its length. The resulting combined sample grade was then displayed, and the outcomes are presented in Fig 3. The grade information from the combined sample was also exported as a text file comprising spatial coordinates alongside the sample grade.

(2) Algorithm design

The MATLAB platform was utilized to construct the IDW algorithm. This algorithm encompasses a recording function that tracks the frequency of usage of composite samples and the weight assigned to the sample distance during the grade estimation process. In addition, the calculation function within the algorithm performs fundamental statistical analyses on the grade estimation results, the number of times each sample is used, and the weights assigned to sample distances. The algorithm can incorporate weightings based on the frequency of sample usage and the grade of the combined sample, as well as distance weight and the grade of the combined sample. It provides functionality for performing weighted result statistics on the combined samples and outputting the corresponding results.

(3) Estimating parameter settings

When IDW was utilized for ore grade estimation and cross-validation, the number of samples was 3, 6, 9, 12, and 15. The power of distance was set to 2, and the distance type employed was Euclidean distance.

(4) Results and analysis

After grade estimation, the frequency of sample usage and the weight of sample distance were analyzed. The impact of the spatial positional relationship between the samples and the estimated blocks on the grade estimation results was also explored. The direct comparison and cross-validation methods were employed for comparisons with the new method. The pros and cons of the weighting based on sample usage frequency and distance were then analyzed. A more thorough analysis was also conducted to identify the issues and solutions encountered in evaluating the effectiveness of the sample-weighted grade estimation method.

### Analysis of sample usage

**Fig 5:**
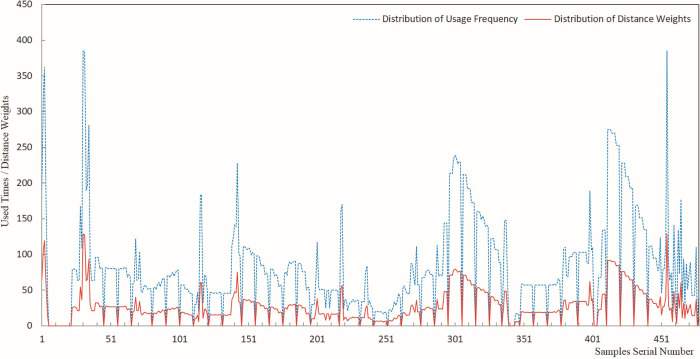
Sample usage results for three samples.

**Fig 6:**
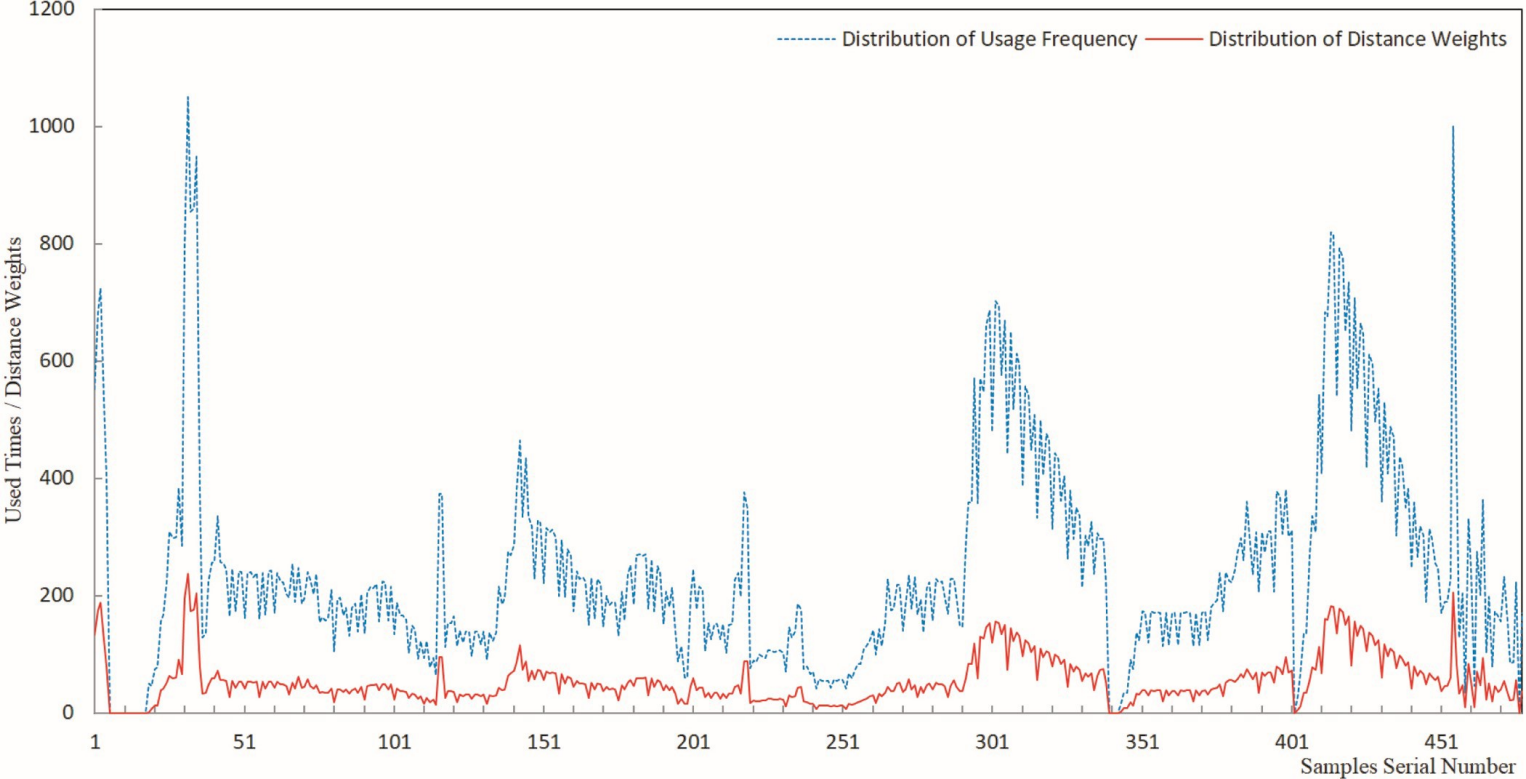
Sample usage results for six samples.

**Fig 7:**
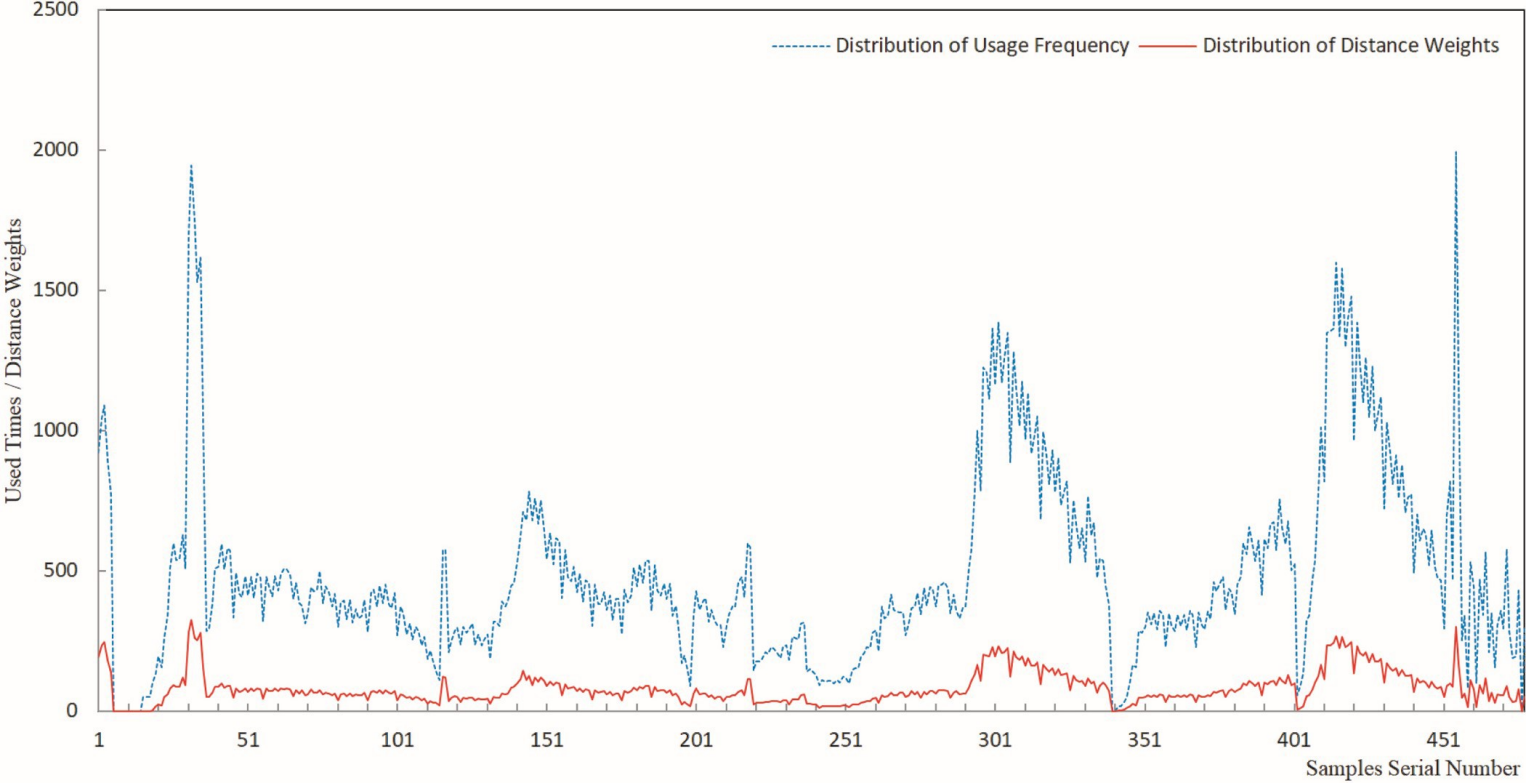
Sample usage results for nine samples.

**Fig 8:**
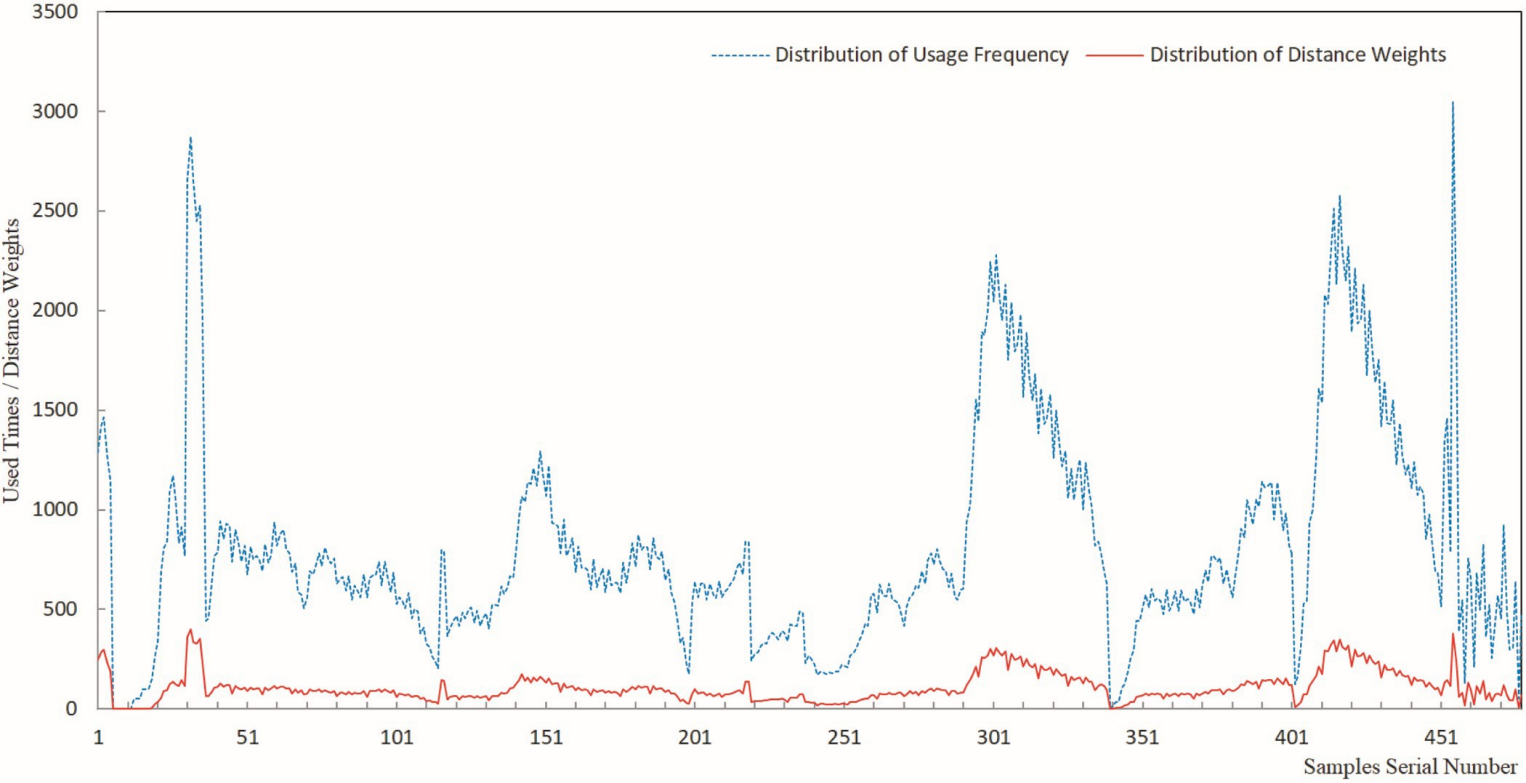
Sample usage results for 12 samples.

**Fig 9:**
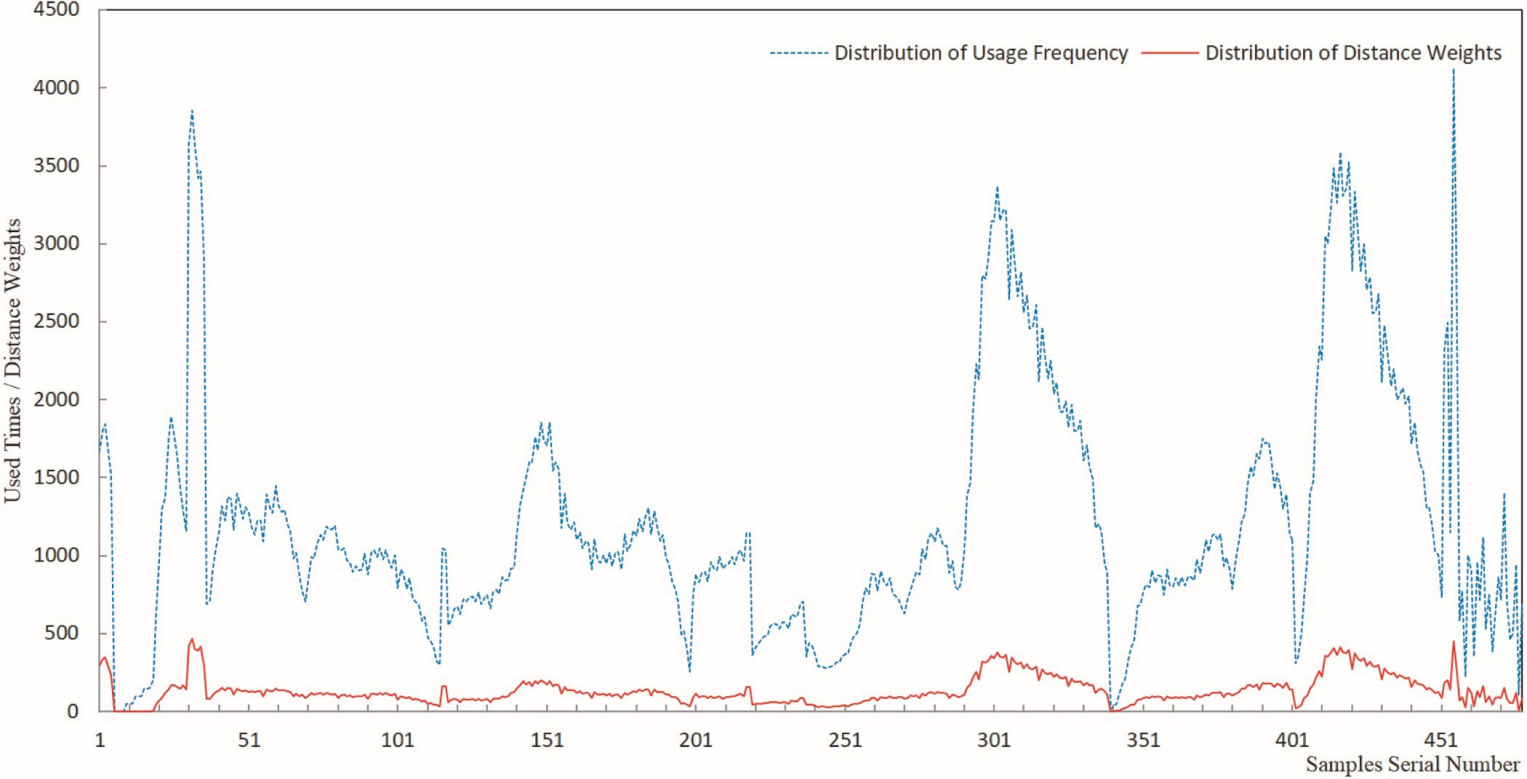
Sample usage results for 15 samples.

The results of sample usage frequency and sample distance weight were analyzed by plotting line charts, offering a visual representation of the estimated results for each sample usage. Figs 5–9 present a comprehensive record of the relevant analysis data. Fig 5 illustrates the recorded results of sample usage frequency and sample distance weight when three Fe grade samples are utilized to estimate a single block. These analyses are a crucial foundation for further investigating the influence of sample usage efficiency and distance weight on estimation accuracy.

In Fig 5, the horizontal axis represents the sequential number of samples during the statistical analysis, while the vertical axis denotes the cumulative count of sample usage and the summation of its distance weights. The trends of sample usage frequency and sample distance weights exhibit a high degree of concordance. The statistical methods for both types of sample usage differ solely in terms of numerical magnitude. Both approaches effectively mirrored the utilization of samples in the grade estimation. The data on sample usage reveal that the utilization of samples is highly uneven. Therefore, theoretically, it is more scientific to assign weights to sample grades based on their usage and compare them with the estimated orebody grades. The sample distance weight is considered the frequency of sample usage, and the spatial distance between the sample and the block is estimated. Thus, theoretically, assigning weights to sample grades based on the sample distance weight is more scientifically rigorous than relying solely on the number of sample uses.

As demonstrated in Figs 5–9, during the estimation of the orebody Fe grade, there was a high degree of consistency in the trends observed in the frequency of sample usage and the sample distance weights. Although the calculation method for sample distance weights theoretically incorporated a more specific consideration of spatial location, both approaches effectively reflected the characteristics of uneven sample usage that resulted from spatial position changes between Fe samples and the blocks to be estimated.

As seen in the details of sample usage frequency and sample distance weights shown in Figs 5–9, there is a notable numerical difference between the two metrics. The variation in sample usage frequency is greater than the sample distance weights. Hence, sample usage frequency is a more effective indicator in revealing the differential characteristics of sample utilization during the estimation process. This also demonstrates that it is unscientific to compare the estimated orebody grade only with sample grades and consider the estimation parameters that yield the closest estimated grade to the sample grade as optimal.

Figs 5–9 indicate that as the number of samples participating in estimating a single block increases from 3 to 15, the frequency of sample usage also rises significantly, along with a notable increase in the sum of the sample distance weights. However, this increase in usage frequency and the sum of weights is not proportional to the increase in the number of samples used to estimate a single block. The utilization of samples was determined by the spatial relationship between the samples and the block being estimated.

Figs 5–9 illustrate the utilization of samples in estimating the Fe grade, revealing significant differences in sample usage frequency. These disparities are often overlooked in routine grade estimation procedures. Hence, examining how sample distribution and orebody spatial morphology affect the estimated grade is crucial. This emphasizes the importance of devising methodologies to tackle this issue.

### Standardization method for sample grade weight

The grade of each sample was weighted by the sum of the usage frequency of individual samples and the distance weights assigned to them. Prior to the weighting process, it was necessary to standardize the sum of sample usage frequencies and sample distance weights. In this study, the Z-score normalization method was first applied, followed by the range normalization method.

(1) Z-score normalization method:

$${\text{W}}_{\text{1}i}=\frac{\lambda_{i}-mean(\lambda_{i})}{Std(\lambda_{i})}$$ (5)

(2) Range normalization method:

$$W_{2i}=\frac{W_{1i}-Min(W_{1})}{Max(W_{1})-Min(W_{1})}$$ (6)

The Z-score and range normalization methods were employed to standardize the sample usage frequencies and sample distance weights, resulting in normalized weights for sample usage frequency (NW) and sample distance (WW). These NW and WW were then employed to weigh the grades of the combined samples.

### Estimation results of the Fe grade of the orebody

Table 2 lists the statistical results of the estimated grades, cross-validation results, and sample grade weighting outcomes following the application of the IDW estimation method. The statistical results of the estimated grades are from the outcomes derived from the IDW estimation method. The cross-validation estimation statistics represent the compiled results of grade estimations at each sample location during the IDW estimation process. The weighted statistical results are based on sample usage frequency and sample distance weights, and the grade statistics rely on the grade statistics after weighting the sample grades according to the number of times a sample was used and its distance weight. The statistical information includes the minimum, maximum, mean, standard deviation, abundance, and skewness of the estimated grades compared to the sample grades. The sample count represents the number of samples involved in the grade estimation of a single block.

**Table 2:** Results of orebody grade estimation and weighted sample grade.

The type of result	Number of samples	Minimum	Maximum	Mean	Standard Deviation	Kurtosis	Skewness
Estimated grade	3	0.2813	1.8548	0.9333	0.2543	4.0149	1.0455
	6	0.3420	1.8336	0.9317	0.2421	3.2703	0.8866
	9	0.4021	1.7373	0.9309	0.2374	2.8582	0.8210
	12	0.4530	1.6902	0.9281	0.2285	2.5292	0.7416
	15	0.4954	1.6497	0.9253	0.2213	2.4040	0.7181
Cross-validation estimation statistics	3	0.3015	1.9312	0.8920	0.2328	5.2658	1.2292
	6	0.3588	1.8878	0.8905	0.2264	4.9707	1.1950
	9	0.4244	1.8062	0.8899	0.2213	4.4816	1.1345
	12	0.4678	1.7557	0.8898	0.2169	4.2058	1.1123
	15	0.5012	1.7018	0.8890	0.2130	4.0363	1.1038
CS+NW	3	0.2325	2.2769	0.8797	0.2701	6.0887	1.4909
	6	0.2321	2.2490	0.8875	0.2723	5.8877	1.4679
	9	0.2321	2.2963	0.8921	0.2744	5.9772	1.4826
	12	0.2326	2.3036	0.9001	0.2778	5.8713	1.4724
	15	0.2335	2.3551	0.9105	0.2827	5.8999	1.4756
CS+WW	3	0.2301	2.2774	0.8796	0.2700	6.0923	1.4909
	6	0.2330	2.2585	0.8859	0.2715	5.8990	1.4656
	9	0.2333	2.3033	0.8938	0.2758	5.9232	1.4765
	12	0.2338	2.3195	0.9016	0.2798	5.8551	1.4741
	15	0.2344	2.3525	0.9087	0.2833	5.8655	1.4775

### Evaluation of the effectiveness of orebody grade estimation

The method to calculate the deviation of the estimated Fe grade is as follows:

$$D_{IDW}=-\frac{P_{IDW}-P_{Sam}}{P_{Sam}}\times 100\%$$ (7)

where *D*_*IDW*_ is the estimation deviation between the statistical quantity of the estimated grades and the statistical quantity of the sample grades; *P*_*IDW*_ is the statistical quantity of the estimated grades; *P_Sam_* corresponds to the statistical quantity of the sample grades.

**Fig 10:**
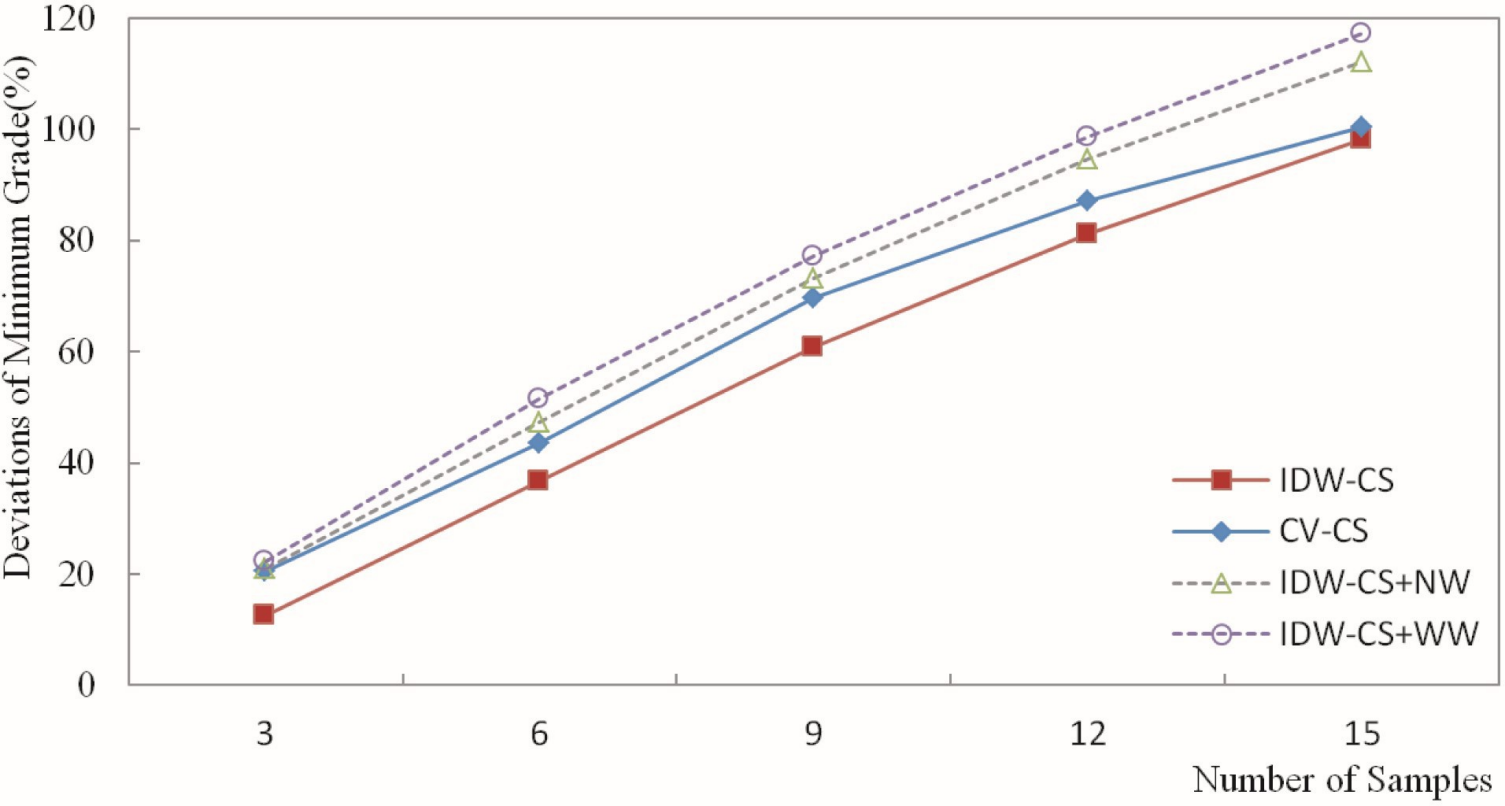
Deviation of the estimated minimum grade.

**Fig 11:**
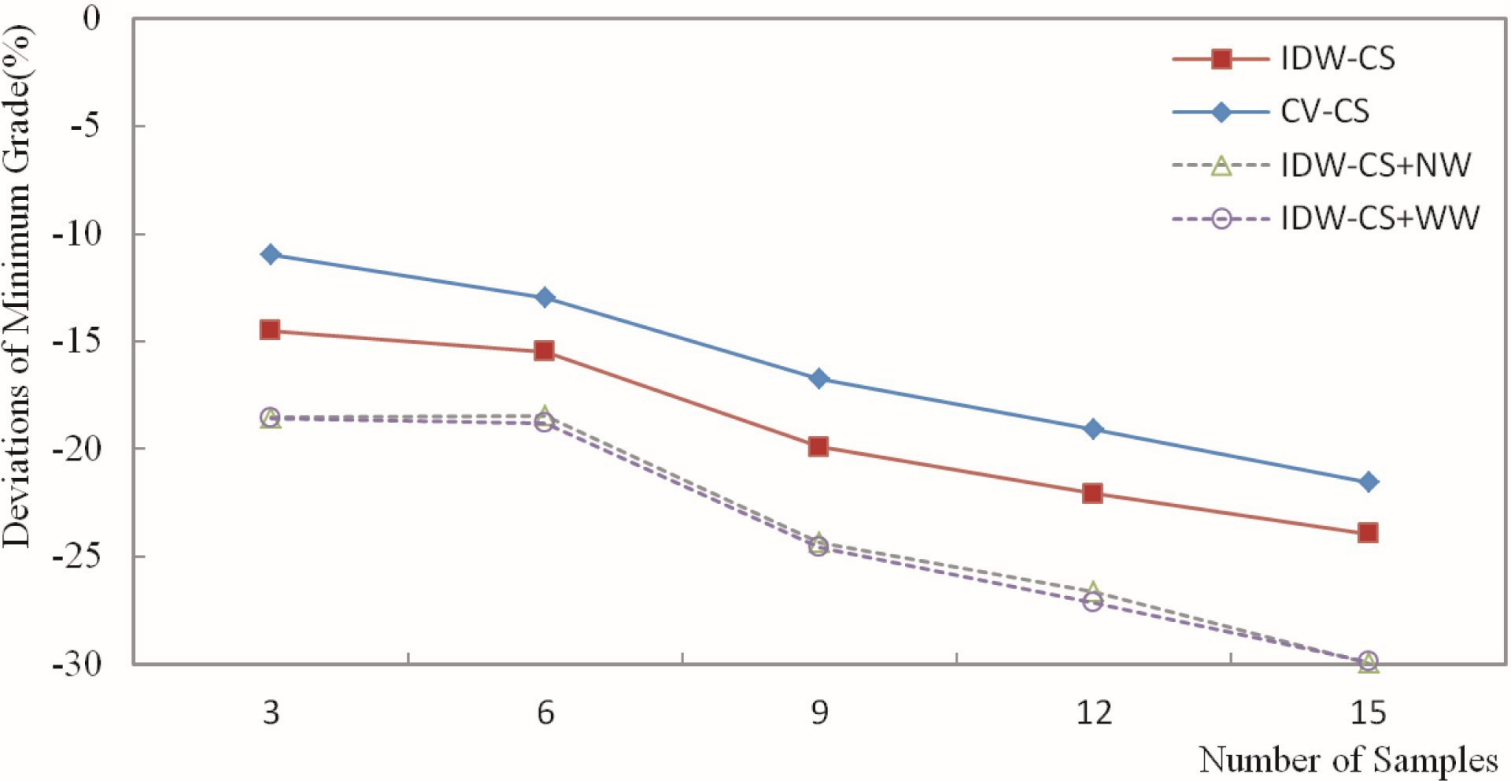
Deviation of the estimated maximum grade.

**Fig 12:**
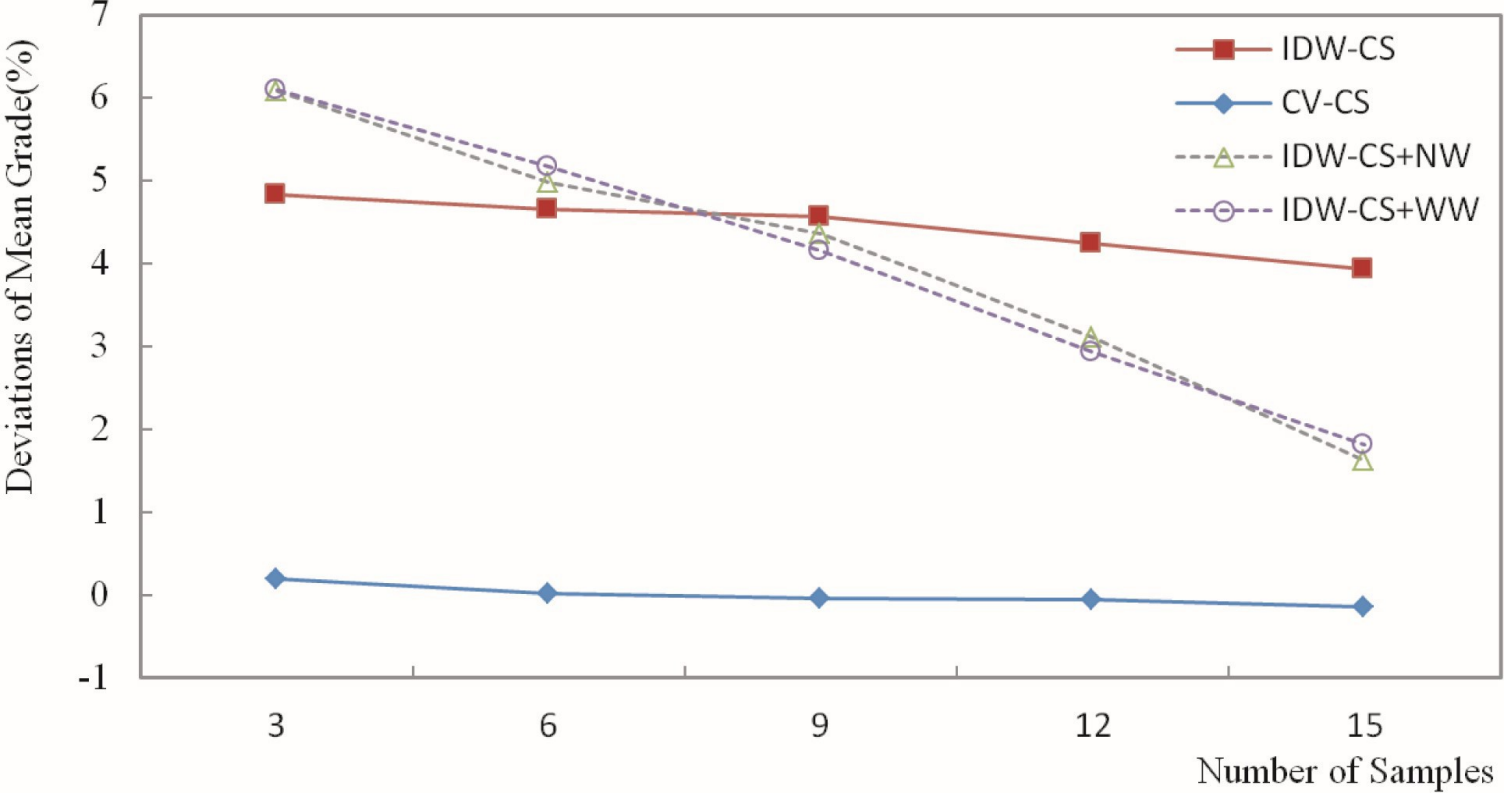
Deviation of estimated average grade.

**Fig 13:**
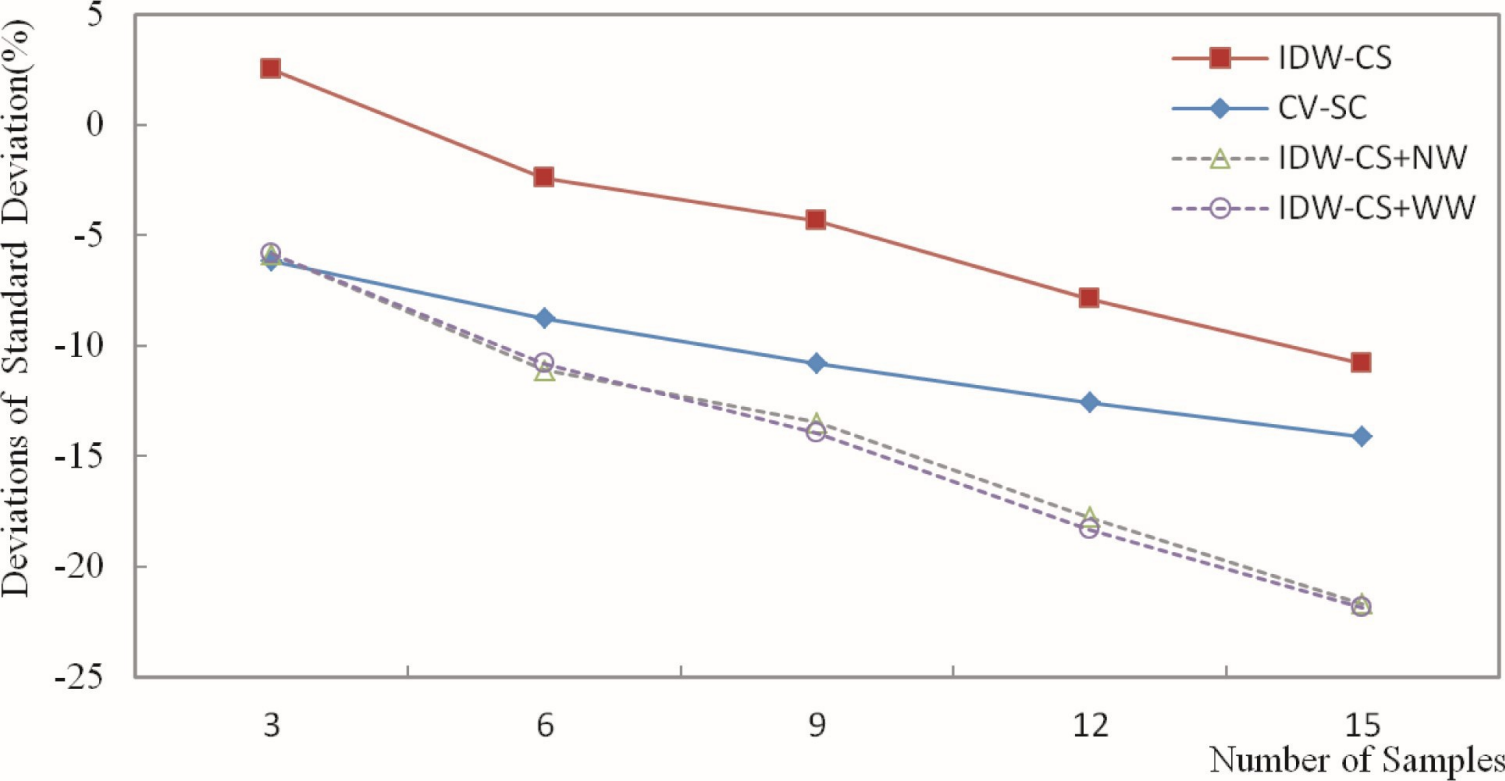
Deviation of estimated grade standard deviation.

The deviation of the estimated grade was calculated based on the estimation of the Fe grade for blocks and the weighting of grades for the composite samples. Then, the deviation results were plotted in figures. Figs 10–13 depict the deviation of the minimum, maximum, mean, and standard deviation of the estimated grades, respectively.

Fig 10 presents the deviation of the minimum estimated grade. It indicates that the horizontal axis represents the number of sample points involved in the estimation, while the vertical axis represents the magnitude of the grade estimation deviation. In Fig 10, IDW-CS denotes the deviation between the minimum grade estimated by IDW and the minimum grade of the composite samples. CV-CS represents the deviation between the minimum grade estimated through cross-validation using IDW and the minimum grade of the composite samples. IDW-CS+NW indicates the deviation between the minimum grade estimated by IDW and the minimum grade of the composite samples weighted by the number of sample usages. IDW-CS+WW denotes the deviation between the minimum grade estimated by the IDW method and the minimum grade of the composite samples weighted by the sample distance weights. Similar representations, as in Fig 10, are used in Figs 11–13.

As indicated in Fig 10, the deviation of the minimum value ranged from 12.53% to 117.28%. The four methods, IDW-CS, CV-CS, IDW-CS+NW, and IDW-CS+WW, exhibited high consistency regarding the minimum estimated grade deviation. Among them, the IDW-CS method yielded the smallest minimum grade deviation, while the IDW-CS+WW method yielded the largest. The deviation of the estimated minimum grade also increased as the number of participating samples increased.

Fig 10 reveals that the minimum deviation of IDW-CS is the smallest, indicating that the deviation of the minimum grade using the direct comparison method is relatively smaller and does not differ significantly from that of DW-CS+NW and IDW-CS+WW. The minimum grade deviations of DW-CS+NW and IDW-CS+WW are relatively similar. The overall trend analysis of the minimum deviation shows that the minimum deviations obtained by IDW-CS+NW and IDW-CS+WW are stable and reliable, demonstrating the feasibility and effectiveness of these two methods.

Fig 11 shows that the deviation of the maximum value ranged from -10.97% to -29.95%. When different numbers of samples were used for the estimation, the CV-CS and IDW-CS+WW methods exhibited the smallest and largest deviation, respectively, in the maximum grade. The four methods, IDW-CS, CV-CS, IDW-CS+NW, and IDW-CS+WW, demonstrated a high degree of consistency in terms of the absolute deviation of the estimated maximum grade, which increased as the number of samples involved in the estimation increased. The trends exhibited by DW-CS+NW and IDW-CS+WW were consistent and closely aligned in deviation values, ranging from a minimum of 0.019% to a maximum of 0.502%. The maximum deviation of CV-CS shows that the cross-validation method had a relatively smaller deviation in the maximum grade. The overall trend of the maximum deviation shows that those determined by IDW-CS+NW and IDW-CS+WW were stable and reliable. The close similarity between IDW-CS+NW and IDW-CS+WW indicates a high degree of similarity when the sample usage frequency and distance weighting are used, as evident from the patterns mentioned earlier and depicted in Figs 5–9.

As depicted in Fig 12, the mean grade deviation ranged from -0.15% to 18.56%. Analyzing the deviation in the estimated mean grade offers an overall indication of the effectiveness of the Fe grade estimation. When different numbers of samples were used for the estimation, the CV-CS method achieved the smallest deviation in the mean grade, while the IDW-CS+WW method resulted in the largest deviation. The mean deviation of CV-CS was significantly smaller than that of the other methods, approaching zero.

This observation likely stems from the consistent proximity of a sample to the block being estimated for orebody grade when utilizing the IDW method for cross-validation. Because the weight of a sample is inversely related to its distance, a sample in close proximity carries significantly more weight than samples located farther away. Consequently, the estimated grade closely resembles that of the nearest sample point.

The mean deviations of IDW-CS, IDW-CS+NW, and IDW-CS+WW decreased as sample points increased. The decrease in mean grade deviation was relatively faster for IDW-CS+NW and IDW-CS+WW; their mean deviations were quite similar. The overall trend of the maximum deviation indicates that the maximum deviations determined by IDW-CS+NW and IDW-CS+WW are stable and reliable. This also demonstrates the similarity between sample usage frequency and distance weighting.

The differences in standard deviation reflect the overall distributional discrepancies between the estimated and sample grades. The deviation in standard deviation characterizes the differences in the distribution patterns between the estimated and sample grades. As shown in Fig 13, the standard deviation ranges from -21.85% to 2.49%. The four methods exhibit generally consistent trends in the deviation of standard deviation, with IDW-CS having a relatively smaller deviation compared to the others, while IDW-CS+NW and IDW-CS+WW are relatively close to each other. Figs 10–13 show that the various estimation biases of IDW-CS+NW and IDW-CS+WW are mutually similar, demonstrating the feasibility and stability of using sample usage frequency and sample distance weights for grade estimation through weighted methods.

## Discussion

(1) As seen in the sample usage patterns shown in Figs 5–9, there were notable differences in how samples were utilized during grade estimation. These differences arose from the varying distribution locations of the samples. This is potentially a crucial factor contributing to the systematic bias observed between the statistical measures of the estimated and sample grades. Therefore, directly comparing estimated grades with sample grades to assess estimation effectiveness is not scientifically sound or reasonable. Consequently, we propose a more scientific and rational approach, initially weighting the sample data based on sample grades and then comparing the estimated grades with the weighted sample grades to evaluate the effectiveness of the estimation. Theoretically, this method offers a more scientific and reasonable means of assessment.
(2) The assessment of ore body grade estimation performance indicates that the four approaches of IDW-CS+NW, IDW-CS+WW, IDW-CS, and CV-CS, while to some extent mutually corroborating each other’s accuracy, resemble the mutual validation among multiple grade estimation methods employed in the literature [6,7]. This study directly compares the estimation results with sample grades in the IDW-CS and CV-CS methods, similar to the methodology adopted in the literature [2,3]. However, comparing numerical sample grades poses challenges in controlling the precision of the comparison outcomes. Maleika and Mahdi, et al [10,13] utilized Root Mean Square Error (RMSE) or Coefficient of Determination (R^2^) to evaluate grade estimation effectiveness. Although RMSE and R^2^ parameters examine the deviation between estimated and sample grades, with optimal results indicated by the minimum statistical values, they fail to account for grade estimation deviations arising from the estimation methods or sample spatial distributions. In contrast, IDW-CS+NW and IDW-CS+WW can overcome the estimation deviations stemming from the IDW estimation method and sample spatial distributions.
(3) The IDW-CS+NW and IDW-CS+WW results demonstrate that the method of sample grade weighting, as an evaluation approach for grade estimation effectiveness, is stable and reliable. From a theoretical perspective, IDW-CS+NW and IDW-CS+WW utilized distance weights to weight the sample grades, thus addressing discrepancies in sample usage stemming from uneven sample distribution. This approach offers a novel means of comparing estimated grades with sample grades. Figs 10–13 indicate that the comparison of IDW-CS+NW and IDW-CS+WW with IDW-CS and CV-CS confirms the stability and reliability of IDW-CS+NW’s and IDW-CS+WW’s evaluation results. The method of weighted statistical analysis of sample grades can be employed to compare sample grades with estimation results, helping to determine the effectiveness of grade estimation.
(4) In theory, the weighted sample grades overcame the uneven usage caused by the spatial relationship between samples and the estimation blocks. This addressed the problem of systematic bias in the statistical results of the estimated sample grades caused by the spatial distribution of ore grades. Therefore, when compared, a closer resemblance between the estimated grades and the weighted sample grades suggests a more accurate estimation. However, when compared with direct comparison methods and cross-validation techniques, the weighted sample grades exhibited a greater discrepancy with the statistical results of IDW (as shown in Figs 10–13). Previous research (in the literature [2,3]) has identified that the magnitude of grade estimation deviation is influenced by the grade distribution of samples and the spatial positional relationship between the estimable blocks and the sampling locations. The current study reveals that the grade estimation deviation is also subject to the inherent bias of the IDW method, with difficulties in quantifying the extent of this deviation. This additional layer of complexity exacerbates the challenge of evaluating the effectiveness of grade estimation. As a biased grade estimation technique, IDW inherently introduces grade estimation deviation. Therefore, exploring the factors that influence the magnitude of IDW estimation bias and conducting quantitative research on IDW estimation deviation is paramount for evaluating grade estimation performance.

## Conclusion

The research aims to explore a more scientific and rational approach for evaluating the effectiveness of ore grade estimation to overcome biases arising from directly comparing estimated grades with sample grades in traditional methods. Through an in-depth analysis of sample utilization patterns and their disparities in spatial distribution, the study proposes an evaluation methodology based on sample grade weighting and then validates its stability and reliability. The key findings can be drawn as follows:

In the process of ore grade estimation, significant variations exist in the utilization samples due to the differing locations of sample distributions. This discrepancy is a pivotal factor contributing to systematic deviations between estimated and sample grades. Hence, a direct comparison of the two to assess estimation effectiveness lacks scientific rigor. The study introduces an innovative evaluation approach, which initially involves weighting sample data based on their grades and comparing the weighted sample grades with the estimated grades to assess estimation effectiveness. Validation using IDW-CS+NW and IDW-CS+WW demonstrates this weighted evaluation method’s theoretical stability and reliability. By incorporating distance weights, these methods balance the impact of uneven sample distribution on grade estimation, providing a novel perspective for comparing estimated grades to sample grades.

This research theoretically enriches the methodological framework for evaluating the effectiveness of ore grade estimation and provides a more scientific evaluation tool for grade estimation in practical mining operations. By statistically analyzing sample grades with weighting, the accuracy of estimation results can be precisely evaluated, guiding mining decision-making and enhancing resource utilization efficiency. Although significant progress was made in evaluating the effectiveness of ore grade estimation in this study, certain limitations remain. Primarily, the research is validated based on specific datasets, and future studies can extend the validation to datasets from diverse geological conditions, ore types, and mining environments to verify the method’s universality. In addition, the influencing factors and bias measurement methods associated with the estimation bias of the IDW method require further investigation to enhance the accuracy and efficiency of the evaluation.

## Supporting information

S1 File (ZIP)
